# Prebiotic intake reduces the waking cortisol response and alters emotional bias in healthy volunteers

**DOI:** 10.1007/s00213-014-3810-0

**Published:** 2014-12-03

**Authors:** Kristin Schmidt, Philip J. Cowen, Catherine J. Harmer, George Tzortzis, Steven Errington, Philip W. J. Burnet

**Affiliations:** 1 Department of Psychiatry, University of Oxford, Warneford Hospital, Oxford, OX3 7JX UK; 2 Clasado Research Services Ltd, Reading, RG6 6BZ UK; 3 Institute for Ageing and Health, Newcastle University, Campus for Ageing and Vitality, Newcastle upon Tyne, NE4 5PL UK

**Keywords:** Cortisol, Hypothalamic-pituitary-adrenal axis, Gut microbiota, Prebiotics, Anxiety, Attention, Emotional processing

## Abstract

**Rationale:**

There is now compelling evidence for a link between enteric microbiota and brain function. The ingestion of probiotics modulates the processing of information that is strongly linked to anxiety and depression, and influences the neuroendocrine stress response. We have recently demonstrated that prebiotics (soluble fibres that augment the growth of indigenous microbiota) have significant neurobiological effects in rats, but their action in humans has not been reported.

**Objectives:**

The present study explored the effects of two prebiotics on the secretion of the stress hormone, cortisol and emotional processing in healthy volunteers.

**Methods:**

Forty-five healthy volunteers received one of two prebiotics (fructooligosaccharides, FOS, or Bimuno®-galactooligosaccharides, B-GOS) or a placebo (maltodextrin) daily for 3 weeks. The salivary cortisol awakening response was sampled before and after prebiotic/placebo administration. On the final day of treatment, participants completed a computerised task battery assessing the processing of emotionally salient information.

**Results:**

The salivary cortisol awakening response was significantly lower after B-GOS intake compared with placebo. Participants also showed decreased attentional vigilance to negative versus positive information in a dot-probe task after B-GOS compared to placebo intake. No effects were found after the administration of FOS.

**Conclusion:**

The suppression of the neuroendocrine stress response and the increase in the processing of positive versus negative attentional vigilance in subjects supplemented with B-GOS are consistent with previous findings of endocrine and anxiolytic effects of microbiota proliferation. Further studies are therefore needed to test the utility of B-GOS supplementation in the treatment of stress-related disorders.

## Introduction

The adult human gut microbiota comprises over 1000 species and 7000 bacterial strains and is characterised by a balanced compositional signature with moderate inter-individual variability (Gareau et al. 2010; Cryan and Dinan 2012). Probiotic strains, which have the ability to confer beneficial effects upon the host, have received renewed attention in recent years (e.g. Forsythe and Kunze 2013). A particular focus has been put on their ability to influence neural and endocrine systems and behavioural phenotypes (Cryan and O’Mahony 2011; Dinan and Cryan 2012). Their potential influence on the mechanisms underlying stress-related disorders such as irritable bowel syndrome (IBS), anxiety and depression is also beginning to be elucidated (Dinan et al. 2006; Rhee et al. 2009; Mayer 2011; Bravo et al. 2012). We have recently demonstrated in rats that prebiotics—oligosaccharides that promote the growth of indigenous beneficial gut bacteria such as *Lactobacilli* and *Bifidobacteria*—also have neurotropic effects (Savignac et al. 2013), but the central actions of these compounds in humans have not been reported.

Convincing evidence now exists for a role of the gut microbiota composition in the regulation of the stress hormone corticosterone (cortisol in humans). Raised levels of circulating corticosterone in germ-free rodents (Crumeyrolle-Arias et al. 2014) are reduced following the administration of probiotics (Sudo et al. 2004), an effect replicated in mice subjected to a stress-inducing behavioural paradigm designed to elevate corticosterone levels (Bravo et al. 2011). However, there is a need to further clarify the mechanisms involved in the complex bidirectional relationship between the stress response and the gut microbiota (Gareau et al. 2007; Dinan and Cryan 2012).

Most indications of a ‘microbiota-gut-brain axis’ in humans have come from patients with gastrointestinal disorders (Brenner et al. 2009; Gareau et al. 2010; Kennedy et al. 2012). Additionally, there is now preliminary evidence for reduced subjective feelings of anxiety and improved aspects of well-being after probiotic intake (Rao et al. 2009; Messaoudi et al. 2011). More recently, a functional MRI investigation found that healthy subjects who received a fermented milk product with probiotics showed decreased BOLD activity to an emotional attention task using facial expressions in the insula and somatosensory regions (Tillisch et al. 2013). These areas play a crucial role in the integration of visceral inputs and the processing of emotional and interoceptive information (Craig 2009). The study demonstrates that manipulations of the gut microbiota can result in measurable changes in emotional processing in the healthy brain.

Neural and behavioural biases in the processing of emotional information, in particular increased processing of threat-related and negatively valenced stimuli, are core functional markers of anxiety and depression (Beck 2008; Cisler and Koster 2010). These markers are evident in symptomatic patients (Sheline et al. 2001) as well as high-risk (Chan et al. 2007) and remitted groups (Bhagwagar and Cowen 2007), and are essential to our understanding of disease symptomatology and treatment efficacy (Harmer et al. 2009; Elliott et al. 2011). Notably, the extent to which these biases can be modulated by pharmacological therapies in patients has been found to be indicative of treatment response (Sheline et al. 2001; Pizzagalli 2010), and assessing novel compounds on their ability to target emotional biases may thus provide a first line of assessing potential clinical utility.

Our study explored the effects of two commercially available prebiotics (fructooligosaccharides [FOS] and Bimuno®-galactooligosaccharides [B-GOS]) on the processing of emotional information and hypothalamic-pituitary-adrenal (HPA) axis activity in healthy human volunteers.

## Materials and methods

### Participants

Forty-five volunteers (22 males, 23 females) recruited through online and poster adverts completed the study. Inclusion criteria were aged 18–45 years, fluent English speaker and BMI range 18–25. Exclusion criteria were previous or current neurological, psychiatric, gastrointestinal or endocrine disorders, or other relevant medical history; current or recent (<3 months) regular medication use; previous or current substance/alcohol dependence or abuse within the last 3 months; regular tobacco use (>5 cigarettes/day); and participation in research studies involving medication intake (within 3 months) or prior completion of the Emotional Test Battery (ETB). To ensure that the enteric environment was consistent in all volunteers, additional exclusion criteria were: no antibiotic use 3 months prior to the study, no regular use of pre- and probiotics (and within 3 months prior to the study) and no vegan diets. Finally, participants were asked to adhere to their regular diets and avoid supplements or special diets. No significant dietary variations were noted.

Participants were assessed with the Structured Clinical Interview for DSM-IV (SCID; First et al. 1997) to confirm the absence of DSM-IV axis I psychiatric conditions. The study was approved by the Oxford Central University Research Ethics Committee. All participants provided written informed consent and were reimbursed for their time and expenses.

### Materials

#### Demographic and questionnaire measures

Participants completed the National Adult Reading Test (NART; Nelson 1982) to provide an estimate of verbal IQ. Self-report questionnaires assessing trait measures of personality (Eysenck Personality Questionnaire, EPQ; Eysenck and Eysenck 1975), stress responsivity (Perceived Stress Reactivity Scale, PSRS; Schlotz et al. 2011), subclinical symptoms of depression (Beck Depression Inventory, BDI; Beck et al. 1961) and anxiety (State-Trait Anxiety Inventory, STAI-trait; Spielberger et al. 1970) were also completed. Before and after prebiotic/placebo intake (days 0 and 21, respectively), anxiety (STAI-state) and measures of perceived stress (Perceived Stress Scale, PSS; Cohen et al. 1983) and mood (Visual Analogue Scales, VAS; Bond and Lader 1974; Positive and Negative Affect Schedules, PANAS; Watson et al. 1988) were measured using self-report questionnaires. The digit span index of verbal working memory was used in order to monitor group differences in executive functioning on the final day of treatment.

#### Prebiotic supplements

The study was placebo controlled, and male/female participants were randomised to receive one of two prebiotics (fructooligosaccharides [*N* = 15; 8 males, 7 females] or Bimuno®-galactooligosaccharides [*N* = 15; 7 males, 8 females]) or a placebo (maltodextrin [*N* = 15; 7 males, 8 females]). The use of maltodextrin as a placebo compound in prebiotic trials is well established (Vulevic et al. 2008). Preparations were provided by Clasado Research Services Ltd., Reading, UK. Participants took the supplements (at 5.5 g per day) in powder form orally with breakfast for 3 weeks. The study used a double-blind randomised design with both the participant and the experimenter being unaware of the group they had been allocated to.

#### Salivary cortisol

HPA axis activity was assessed on the day before (day 0) and on the final day of prebiotic/placebo administration (day 21), using the salivary cortisol awakening response (CAR; Pruessner et al. 1997). For each CAR measurement, participants were instructed to provide five saliva samples (using Salivettes, Sarstedt Ltd., Nümbrecht, Germany) taken in their own home immediately upon waking and subsequently every 15 min until 1 h post-waking. Saliva samples were stored at 4 °C prior to analysis. Cortisol was measured using a commercial ELISA (Salimetrics Europe Ltd., Newmarket, UK), within 7 days of sample collection.

#### Emotional processing tasks

On the final day of prebiotic/placebo intake (day 21), participants completed a validated computerised test battery assessing the processing of emotional stimuli (the ETB; Harmer et al. 2004).

#### Attentional dot-probe task

Sixty negative and 60 positive words were paired with neutral words matched for length. On each trial, a fixation cross was presented for 500 ms in the centre of the screen, followed by two words presented at the top and bottom of the screen. In the unmasked condition, the words were presented for 500 ms. In the masked condition, word pairs were presented for 17 ms after which a mask was displayed for 483 ms. Masks were constructed from digits, letters and non-letter symbols and were matched for word position and length. Words or masks were replaced by a probe of either one or two stars in the location of one of the preceding stimuli (probes were presented at the top or bottom of the screen with equal frequency). Participants were instructed to indicate the number of stars as quickly and accurately as possible using two labelled keys. A key press terminated the probe presentation and trial. There were 180 trials in total (30 positive-neutral, 30 negative-neutral, 30 neutral-neutral word pairs each for masked and unmasked conditions), and emotional words were presented at the top and the bottom location with equal frequency. Masked and unmasked trials were presented in random order. Reaction time and accuracy scores were recorded, and attentional vigilance scores were calculated for each participant by subtracting the reaction time from trials when probes appeared in the same position as the emotional word (congruent trials) from those trials when probes appeared in the opposite position to the emotional word (incongruent trials).

#### Facial expression recognition task

In the facial expression recognition task (FERT), the perception of six basic emotions (happiness, surprise, sadness, fear, anger, disgust) or a neutral expression (taken from the Pictures of Affect Series; Ekman and Friesen 1976) was assessed. Each emotion was shown at 10 morphed intensity levels from neutral to maximum emotional expression (Young et al. 1997) leading to a total of 250 randomly presented stimuli. Each stimulus was presented for 500 ms and replaced by a grey screen until the participant responded (as quickly and as accurately as possible) by selecting a key corresponding to one of the basic emotions. The outcome measures were classification accuracy, number of misclassifications and reaction times.

#### Emotional categorisation and memory

Sixty words representing either disagreeable (*N* = 30) or agreeable (*N* = 30) personality characteristics (from Anderson 1968) and matched for meaningfulness, word frequency and word length were presented on a computer screen for 500 ms each. Participants were asked to categorise each word as quickly and accurately as possible according to whether they would like or dislike to be described by it. After completion, participants were instructed to recall and write down as many words as they could within a 2-min time limit. Subsequently, participants were instructed to categorise words presented on the screen into those which were previously presented (60 target words) or those which were novel words (60 matched distracter words). Outcome measures for the emotional categorisation were classifications and reaction times. For the memory recall, the number of correct responses and false positives was recorded, and participants’ memory recognition was assessed using correct responses, false positives and reaction times.

### Statistical analysis

Demographic and questionnaire values were analysed using one-way ANOVA with group as factor. Salivary cortisol values (in nmol/l) were square root transformed and analysed in a mixed design ANOVA with time point of sampling (0, 15, 30, 45 and 60 min after waking) and day of sampling (pre- vs. post-treatment) as repeated-measures variables and prebiotic treatment group (placebo, FOS or B-GOS) as a between-subjects variable. Raw cortisol values are presented for clarity. The behavioural outcome variables of the ETB were also analysed with mixed design ANOVAs, with prebiotic treatment group as between-subjects factor and emotion and task condition as within-subjects factors. Significant interactions were followed up using main effects analyses. Greenhouse-Geisser corrections were used where assumptions of sphericity were not met.

## Results

### Baseline measures and compliance

There were no significant differences between groups for age, trait measures of anxiety, stress reactivity, neuroticism or cognitive status as assessed by digit span (see Table 1). This suggests that groups were well matched between the two prebiotic and placebo conditions.

**Table 1:** Baseline characteristics of participants by treatment group

Measure	Mean (SD)			*p*
	Placebo	FOS	B-GOS	
Age, in years	23.27 (3.86)	24.53 (3.87)	23.27 (3.95)	0.31
NART score	116.50 (5.11)	115.71 (5.24)	112.62 (5.99)	0.18
EPQ, neuroticism	5.17 (4.61)	5.64 (4.22)	4.38 (3.36)	0.73
Perceived Stress Reactivity Scale	16.92 (10.06)	16.86 (6.95)	14.38 (5.66)	0.64
Beck Depression Inventory	2.58 (4.21)	2.14 (3.04)	2.54 (2.76)	0.93
Spielberger Anxiety Inventory, trait	32.00 (10.33)	32.86 (5.41)	31.69 (4.80)	0.91
Digit span, forward	10.08 (1.38)	8.43 (2.31)	8.54 (2.60)	0.12
Digit span, backward	8.50 (1.98)	6.79 (2.52)	7.69 (2.29)	0.18

Out of 48 participants, three participants did not complete the full course of prebiotic/placebo intake and were excluded from all analyses, leading to the final sample of 45 participants. Participants completed a checklist each day to monitor prebiotic/placebo intake, and none reported missing more than two intakes (0 missed = 41, 1 missed = 3, 2 missed = 1).

### Hormonal contraceptive use and menstrual cycle

Of the 23 female participants, 13 used hormonal methods of contraception. Menstrual cycle phase was reported by 16 females, and an additional two female participants reported no or very infrequent menses due to hormonal contraceptive use—these were coded as a separate group. Cycle phase was analysed according to the following phases (based on averages from Wolfram et al. 2011; Fehring et al. 2006): menses (days 1–6), follicular phase (days 7–12), ovulation phase (days 13–19), luteal phase (day 20–end of cycle). In order to test the between-subjects effects of contraceptive use on the waking cortisol response, repeated-measures ANOVAs were performed on the cortisol level upon waking (first sample) and the cortisol area under the curve with respect to ground (AUC-g) including the factor supplement group to test for potential interactions.

There was no significant difference in the number of females who took hormonal contraceptives between the groups (*χ* ^2^ = 3.45, *p* > 0.1). The effect of hormonal contraceptive on the first sample of salivary cortisol after waking showed a trend for lower levels in females taking contraceptives compared to those who were not (mean (SD) = 8.20 (3.42) vs. mean (SD) = 10.65 (4.37), *F*(1,17) = 3.74, *p* = 0.07); however, this difference was stable across days of testing and treatment groups (all interactions’ *p* values >0.7). The effect of hormonal contraceptives on cortisol AUC-g was not significant (*F*(1,17) = 3.01, *p* > 0.1), and there were no significant interactions with testing day or treatment group (all *p* > 0.1).

The number of participants who were in a particular cycle phase at the time of testing did not differ between the groups (*χ* ^2^ = 8.75, *p* > 0.1). Due to insufficient power, the effects of menstrual phase on cortisol or attention were not tested with ANOVAs.

### Cortisol

**Fig. 1:**
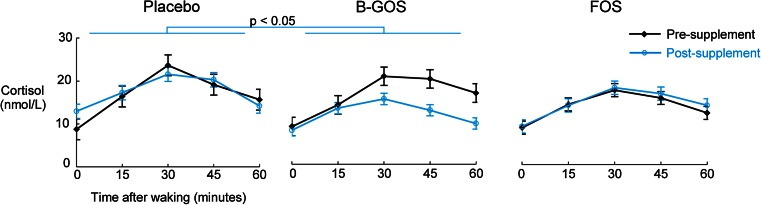
Cortisol awakening response before and after administration of placebo, B-GOS or FOS. There were no differences in the salivary CAR pre-administration. Salivary cortisol awakening response was significantly lower after 3 weeks of B-GOS intake, but not FOS intake, compared with placebo

Salivary cortisol did not differ significantly between groups at baseline but was significantly lower following B-GOS compared with placebo (Fig. 1). This was shown by an ANOVA interaction effect of pre- versus post-treatment, group (placebo, FOS or B-GOS) and sampling time point (in minutes post-waking) on salivary cortisol levels, followed up with separate group × time point ANOVAs for each day of sampling (day 0: main effect of group *F*(2,41) = 1.08, n.s.; day 21: main effect of group *F*(2,41) = 4.20, *p* < 0.05, followed up with Sidak-corrected contrasts: placebo vs. GOS, *p* = 0.02, all others *p* > 0.1). Main effects analyses of the day × group × time ANOVA confirmed that cortisol levels were increased post-waking 15, 30, 45 and 60 min after waking (significant main effect of time *F*(2.41,98.63) = 58.61, *p* < 0.001, planned follow-up contrasts all significant at *p* < 0.001). The main effects of day of sampling and treatment group were not significant (*p* > 0.1). Gender was not entered as a factor of interest due to insufficient power.

**Fig. 2:**
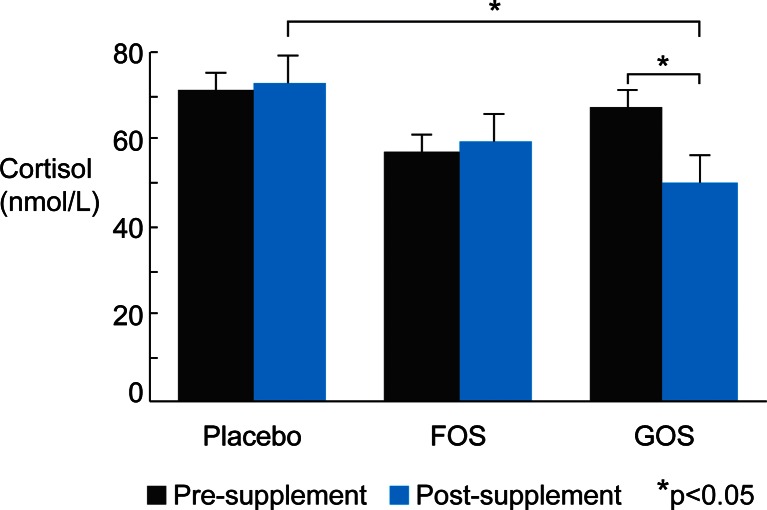
Area under the curve (with respect to ground) of salivary cortisol awakening response pre- and post-prebiotic supplement/placebo intake. *p < 0.05

The lowered CAR in the B-GOS compared to the placebo group on day 21 of supplement administration was also confirmed when analysing area under the curve with respect to ground (Fig. 2; day × group ANOVA on square-root-transformed salivary cortisol values: day × group interaction [*F*(2,41) = 3.52, *p* = 0.039], followed up with separate group ANOVAs for pre/day 0 [*F*(2,41) = 1.24, n.s.] and post/day 21 [*F*(2,41) = 4.12, *p* = 0.023, follow-up contrasts: placebo vs. B-GOS *p* = 0.019, placebo vs. FOS *p* > 0.1, FOS vs. B-GOS *p* > 0.1]).

### Emotional Test Battery

#### Attentional dot-probe task

**Fig. 3:**
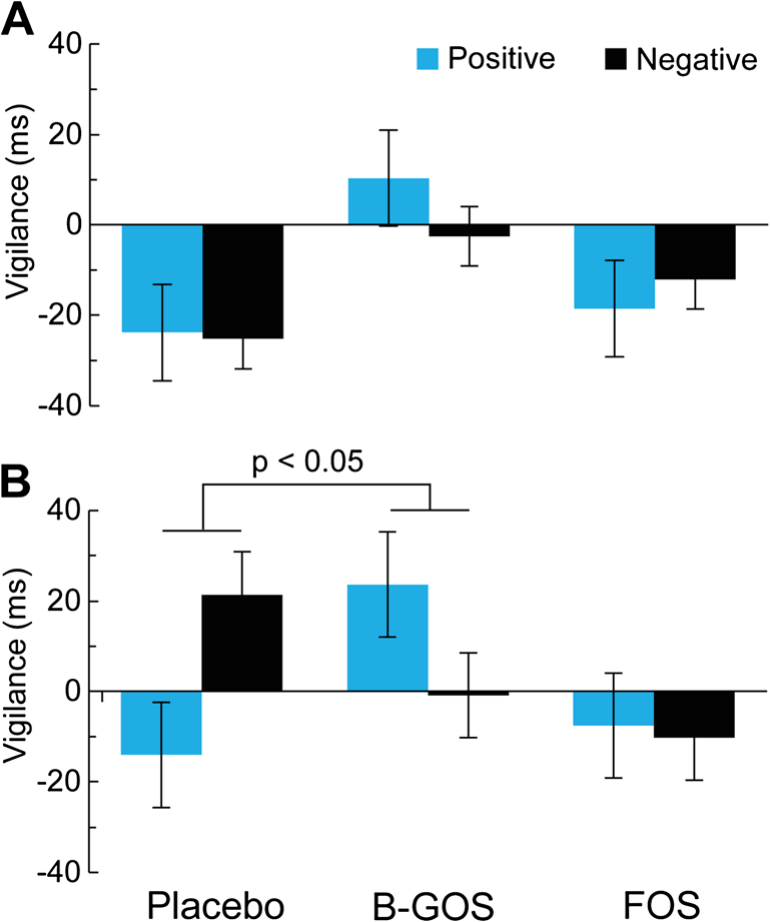
Vigilance reaction times in the attentional dot-probe task. **a** Attentional vigilance did not differ between groups during masked trials of the attentional dot-probe task. **b** Participants showed decreased attentional vigilance to negative versus positive words in the unmasked condition of the dot-probe task after B-GOS but not FOS intake compared to placebo

There was a significant group × emotion × masking condition interaction in the visual dot-probe task (group × emotion × masking condition [*F*(2,41) = 3.14, *p* = 0.05]). As can be seen in Fig. 3, this effect was driven by decreased attentional vigilance to negative versus positive information in the unmasked condition (Fig. 3b), with no significant main effects or interactions in the masked condition (Fig. 3a; valence × group interaction in unmasked: *F*(2,41) = 4.29, *p* = 0.02; masked: *F*(2,41) = 0.85, *p* > 0.1). Follow-up analyses with separate ANOVAs for prebiotic group compared with placebo in the unmasked condition confirmed this effect as driven by increased positive versus negative vigilance after B-GOS compared to placebo, while the FOS group did not perform differently to placebo (B-GOS vs. placebo: valence × group *F*(1,27) = 6.94, *p* = 0.014, FOS vs. placebo: valence × group *F*(1,27) = 3.20, n.s.).

#### FERT

There were no significant effects of prebiotic treatment on measures of accuracy (main effect of group: *F*(2,42) = 1.71, n.s., emotion × group interaction *F*(7.83,164.52) = 0.67, n.s.). Analysis of reaction time data revealed no significant interaction between group and emotion (main effect of group: *F*(2,42) = 0.53, n.s.; emotion × group interaction *F*(8.16,773.38) = 1.10, n.s.).

#### Emotional categorisation, recall and recognition

Participants responded faster to positive (mean reaction time = 1031 ms, SD = 228 ms) compared to negative (mean reaction time = 1083 ms, SD = 200 ms) self-referential personality words in the emotional categorisation task (valence × group ANOVA, main effect of valence: *F*(1,42) = 12.82, *p* < 0.01) and in the emotional word recognition task (mean reaction time to positive words = 1220.92, SD = 263.03; mean reaction time to negative words = 1381.07, SD = 348.13; main effect of valence: *F*(1,41) = 23.34, *p* < 0.001); however, there was no significant main effect of prebiotic treatment group (*F*(2,42) = 0.80, *p* > 0.1) and the relative speeding for positive words did not differ between groups (emotion × group *F*(2,42) = 0.35, *p* > 0.1). Positive words were also remembered more often than negative words in both the surprise recall task (mean accuracy, positive words = 7.41 (SD = 2.45), negative = 5.70 (2.29); *F*(1,41) = 16.16, *p* < 0.001) and in the recognition task (mean correct recognition, positive words = 25.67 (SD = 3.41), negative words = 22.36 (SD = 3.88); *F*(1,41) = 53.54, *p* < 0.001), but these effect did not differ between groups (*p* > 0.1).

#### Self-report questionnaires

There were no significant effects of group on self-report measures of state anxiety or perceived stress before or after prebiotic/placebo administration (see Table 2). Furthermore, there were no group differences in the overall cognitive status as assessed by digit span on the day of psychological testing.

**Table 2:** State measures of anxiety and perceived stress (mean, SD) before and after prebiotic/placebo administration and cognitive status on day of assessment (day 21)

Measure		Mean (SD)			*p*
		FOS	Placebo	B-GOS	
Spielberger Anxiety Inventory, state	Pre (day 0)	32.00 (7.13)	32.92 (10.03)	32.15 (9.11)	0.96
	Post (day 21)	30.00 (5.53)	30.17 (7.55)	31.31 (5.23)	0.84
Perceived Stress Scale	Pre (day 0)	9.86 (4.64)	9.92 (6.05)	10.77 (3.77)	0.87
	Post (day 21)	9.57 (5.20)	8.92 (5.40)	10.46 (4.86)	0.75
Digit span (post, day 21)	Forward	9.71 (2.40)	10.25 (1.96)	9.54 (2.44)	0.72
Digit span (post, day 21)	Backward	7.50 (2.82)	8.25 (2.73)	8.62 (2.66)	0.56

### Correlational analyses

To test the hypothesis that cortisol levels were associated with changes in attentional dot-probe performance in the B-GOS group, we correlated difference scores of positive versus negative reaction times in the unmasked condition with absolute cortisol levels upon waking on the day of testing and with the difference in pre- versus post-prebiotics cortisol values (day 0–day 21). There were no associations of cortisol with attentional performance (all *p* > 0.1).

## Discussion

The current study explored the neuroendocrine and affective effects of two types of prebiotic supplements in healthy human volunteers, using salivary CAR and a validated test battery of emotional processing. Results revealed that B-GOS prebiotic intake was associated with decreased waking salivary cortisol reactivity and altered attentional bias compared to placebo. These results are consistent with previously found anxiolytic-like effects of probiotics and reveal key differences between two different prebiotic supplements.

Our findings of lowered cortisol awakening reactivity in the group receiving B-GOS prebiotics compared to the placebo group indicate that prebiotic administration may modulate HPA activity in a similar fashion as the administration of probiotic strains directly seen in rodents (Sudo et al. 2004; Gareau et al. 2007) and humans (Messaoudi et al. 2011). The cortisol awakening response is a reliable marker of HPA axis activity which has been found to be increased by work stressors (Pruessner et al. 1997; Kunz-Ebrecht et al. 2004) and in individuals at high risk of depression (Mannie et al. 2007). Insufficient or excessive cortisol reactivity may indicate dysfunctional HPA axis feedback mechanisms, which may provide useful targets for modulation by treatments in certain vulnerability or disease states (Pariante and Lightman 2008; Dinan and Cryan 2012).

Participants receiving B-GOS supplements showed increased attentional vigilance to positive versus negative stimuli on the dot-probe task. Our effects are similar to those seen following administration of pharmacological agents such as the selective serotonin reuptake inhibitor citalopram or the benzodiazepine diazepam in healthy individuals (Browning et al. 2006; Murphy et al. 2009a, b). These effects have been interpreted as showing an early anxiolytic-like profile, where threatening stimuli are less likely to be attended to (Harmer 2010). Interestingly, we found effects of the B-GOS prebiotic administration on altered attentional processing only in the unmasked condition (500 ms presentation) of the dot-probe task. Attentional vigilance to brief, masked presentations of threatening cues has primarily been interpreted as an involuntary deployment of attention (Browning et al. 2010), whereas at longer stimulus durations, it may further involve a difficulty to disengage from salient emotional stimuli (Koster et al. 2004; Cisler and Koster 2010). Research suggests that while responses to masked stimuli may be particularly prevalent in anxiety disorders rather than depression, attentional bias seen at longer exposure durations may also be of relevance to depression. The increase of positive compared to negative emotional information processing in the B-GOS group provides initial evidence that behavioural effects of probiotics in rodent models (Bravo et al. 2011) can be extended to affective processing in humans using prebiotics. These results are also consistent with a recent fMRI study which reported that a 3-week probiotic administration reduced neural response in a network of areas (including the somatosensory cortex, insula and parahippocampal gyrus) to angry and fearful facial expressions (Tillisch et al. 2013).

Differences in attentional resource allocation to negative or positive stimuli are associated with individual variability in trait and state measures. Specifically, vigilance to cues of threat or danger is greater in highly anxious individuals compared with low-anxious individuals and healthy controls (Mogg et al. 1994; Bradley et al. 1998; Koster et al. 2005), and threat-related processing is believed to play a key role in the symptomatology of anxiety and its modulation by anxiolytics (Beck and Clark 1997; Mogg and Bradley 1998). Pharmacological anxiolytics and antidepressants that are clinically effective in reducing symptoms have been found to affect reductions in specific negative biases in neural correlates of emotional information processing (Sheline et al. 2001; Fu et al. 2004; Godlewska et al. 2012), and also modulate biases when administered in healthy control and at-risk groups (Harmer et al. 2003; Browning et al. 2006; Murphy et al. 2009b). Based on the initial results of gut microbiota interventions, it is now crucial to investigate the extent and specificity of information processing biases that may be targeted by different gut microbiota manipulations and whether they prove clinically beneficial.

Although we were unable to test the mechanisms of action directly through characterisation of gut microbiota, a previous characterisation of B-GOS and FOS prebiotics showed pronounced increases in bifidobacteria in faecal pellets of rats treated with B-GOS administration compared to placebo, with more moderate effects following the FOS intervention (Savignac et al. 2013). The specificity of galactooligosaccharides affecting behavioural and endocrine changes is thus in line with previous findings of galactooligosaccharides as particularly effective in stimulating enteric microbial growth (Abou Hachem et al. 2013). Of course, the possibility of an additional, direct effect of B-GOS on the gut mucosa [10] cannot be ruled out.

Given the lack of association between altered attentional processes and a reduction in the salivary cortisol awakening response (CAR), we were unable to confirm a moderating effect of cortisol reactivity on behaviour. Although there is strong evidence for a role of the gut microbiota in the regulation of the HPA axis (see Dinan and Cryan 2012 for a review), the exact mechanisms by which they interact with central effects remain to be investigated. One potential mechanism of action underlying these effects is via anti-inflammatory and immune responses following probiotic proliferation (Lyte 2011; Ait-Belgnaoui et al. 2012), and central effects of probiotics have been found to be vagus nerve dependent (Bravo et al. 2011).

While initial results of the B-GOS prebiotic on the cortisol awakening response are promising, we had insufficient power to investigate gender effects, which have previously been reported (Pruessner et al. 1997). Further, there were no effects of either prebiotic on the remaining tasks of the ETB which examine aspects of facial expression recognition, self-referential processing and emotional memory. The current results also differ from previous findings that indicate subjective anxiolytic effects of probiotics (Rao et al. 2009; Messaoudi et al. 2011) as we found no effects of prebiotics on subjective measures of subclinical anxiety or perceived stress. One improvement may be to extend the administration period of prebiotics as probiotics take several weeks to proliferate. It is also possible that these results are in part due to our study population of young healthy volunteers with low subclinical scores and presumably healthy gut microbiota compositions even before treatment. Studying a population with a potential deficiency in their gut microbiota compositions, for example elderly individuals or IBS patients with comorbid psychiatric symptoms, may improve power. The applicability of our results to other populations—such as those with deterioration in the health of their intestinal gut flora or HPA axis abnormalities—is a valid next step for future study.

We found a selective modulation of attention to emotional stimuli and HPA axis reactivity following B-GOS prebiotic supplement in healthy participants, supporting a key role for gut microbiota in the regulation of affective function. This, to our knowledge, is the first study extending findings of the central effects of probiotics to behavioural effects of prebiotics in humans.

## References

[CR1] Abou Hachem M, Andersen JM, Barrangou R (2013). Recent insight into oligosaccharide uptake and metabolism in probiotic bacteria. Biocatal Biotransform.

[CR2] Ait-Belgnaoui A, Durand H, Cartier C (2012). Prevention of gut leakiness by a probiotic treatment leads to attenuated HPA response to an acute psychological stress in rats. Psychoneuroendocrinology.

[CR3] Anderson N (1968). Likableness ratings of 555 personality trait words. J Pers Soc Psychol.

[CR4] Beck AT (2008). The evolution of the cognitive model of depression and its neurobiological correlates. Am J Psychiatr.

[CR5] Beck AT, Clark DA (1997). An information processing model of anxiety: automatic and strategic processes. Behav Res Ther.

[CR6] Beck AT, Ward C, Mendelson M (1961). An inventory for measuring depression. Arch Gen Psychiatry.

[CR7] Bhagwagar Z, Cowen PJ (2007). ‘It’s not over when it’s over’: persistent neurobiological abnormalities in recovered depressed patients. Psychol Med.

[CR8] Bond A, Lader M (1974). The use of analogue scales in rating subjective feelings. Br J Med Psychol.

[CR9] Bradley BP, Mogg K, Falla SJ, Hamilton LR (1998). Attentional bias for threatening facial expressions in anxiety: manipulation of stimulus duration. Cogn Emot.

[CR10] Bravo JA, Forsythe P, Chew MV (2011). Ingestion of Lactobacillus strain regulates emotional behavior and central GABA receptor expression in a mouse via the vagus nerve. Proc Natl Acad Sci.

[CR11] Bravo JA, Julio-Pieper M, Forsythe P (2012). Communication between gastrointestinal bacteria and the nervous system. Curr Opin Pharmacol.

[CR12] Brenner DM, Moeller MJ, Chey WD, Schoenfeld PS (2009). The utility of probiotics in the treatment of irritable bowel syndrome: a systematic review. Am J Gastroenterol.

[CR13] Browning M, Reid C, Cowen PJ (2006). A single dose of citalopram increases fear recognition in healthy subjects. J Psychopharmacol.

[CR14] Browning M, Holmes EA, Harmer CJ (2010). The modification of attentional bias to emotional information: a review of the techniques, mechanisms, and relevance to emotional disorders. Cogn Affect Behav Neurosci.

[CR15] Chan SWY, Goodwin GM, Harmer CJ (2007). Highly neurotic never-depressed students have negative biases in information processing. Psychol Med.

[CR16] Cisler JM, Koster EHW (2010). Mechanisms of attentional biases towards threat in anxiety disorders: an integrative review. Clin Psychol Rev.

[CR17] Cohen S, Kamarck T, Mermelstein R (1983). A global measure of perceived stress. J Health Soc Behav.

[CR18] Craig AD (2009). How do you feel—now? The anterior insula and human awareness. Nat Rev Neurosci.

[CR19] Crumeyrolle-Arias M, Jaglin M, Bruneau A (2014). Absence of the gut microbiota enhances anxiety-like behavior and neuroendocrine response to acute stress in rats. Psychoneuroendocrinology.

[CR20] Cryan JF, Dinan TG (2012). Mind-altering microorganisms: the impact of the gut microbiota on brain and behaviour. Nat Rev Neurosci.

[CR21] Cryan JF, O’Mahony SM (2011). The microbiome-gut-brain axis: from bowel to behavior. Neurogastroenterol Motil.

[CR22] Dinan TG, Cryan JF (2012). Regulation of the stress response by the gut microbiota: implications for psychoneuroendocrinology. Psychoneuroendocrinology.

[CR23] Dinan TG, Quigley EMM, Ahmed SMM (2006). Hypothalamic-pituitary-gut axis dysregulation in irritable bowel syndrome: plasma cytokines as a potential biomarker?. Gastroenterology.

[CR24] Ekman P, Friesen WV (1976) Pictures of Facial Affect. Consulting Psychologists Press, Palo Alto

[CR25] Elliott R, Zahn R, Deakin JFW, Anderson IM (2011). Affective cognition and its disruption in mood disorders. Neuropsychopharmacol Off Publ Am Coll Neuropsychopharmacol.

[CR26] Eysenck H, Eysenck S (1975). Manual of the Eysenck personality questionnaire.

[CR27] Fehring RJ, Schneider M, Raviele K (2006). Variability in the phases of the menstrual cycle. J Obstet Gynecol Neonat Nurs.

[CR28] First M, Spitzer R, Gibbon M, Williams J (1997) Structured Clinical Interview for DSM-IV axis I disorders (SCID-I) 10.1001/archpsyc.1992.01820080032005 1637252

[CR29] Forsythe P, Kunze WA (2013). Voices from within: gut microbes and the CNS. Cell Mol Life Sci.

[CR30] Fu CHY, Williams SC, Cleare AJ (2004). Attenuation of the neural response to sad faces in major depression by antidepressant treatment: a prospective, event-related functional magnetic resonance imaging study. Arch Gen Psychiatry.

[CR31] Gareau MG, Jury J, MacQueen G (2007). Probiotic treatment of rat pups normalises corticosterone release and ameliorates colonic dysfunction induced by maternal separation. Gut.

[CR32] Gareau MG, Sherman PM, Walker WA (2010). Probiotics and the gut microbiota in intestinal health and disease. Nat Rev Gastroenterol Hepatol.

[CR33] Godlewska BR, Norbury R, Selvaraj S (2012). Short-term SSRI treatment normalises amygdala hyperactivity in depressed patients. Psychol Med.

[CR34] Harmer CJ (2010). Antidepressant drug action: a neuropsychological perspective. Depress Anxiety.

[CR35] Harmer CJ, Bhagwagar Z, Perrett DI (2003). Acute SSRI administration affects the processing of social cues in healthy volunteers. Neuropsychopharmacology.

[CR36] Harmer CJ, Shelley NC, Cowen PJ, Goodwin GM (2004). Increased positive versus negative affective perception and memory in healthy volunteers following selective serotonin and norepinephrine reuptake inhibition. Am J Psychiatr.

[CR37] Harmer CJ, Goodwin GM, Cowen PJ (2009). Why do antidepressants take so long to work? A cognitive neuropsychological model of antidepressant drug action. Br J Psychiatr J Ment Sci.

[CR38] Kennedy PJ, Clarke G, Quigley EMM (2012). Gut memories: towards a cognitive neurobiology of irritable bowel syndrome. Neurosci Biobehav Rev.

[CR39] Koster EHW, Crombez G, Verschuere B, De Houwer J (2004). Selective attention to threat in the dot probe paradigm: differentiating vigilance and difficulty to disengage. Behav Res Ther.

[CR40] Koster EHW, Verschuere B, Crombez G, Van Damme S (2005). Time-course of attention for threatening pictures in high and low trait anxiety. Behav Res Ther.

[CR41] Kunz-Ebrecht SR, Kirschbaum C, Marmot M, Steptoe A (2004). Differences in cortisol awakening response on work days and weekends in women and men from the Whitehall II cohort. Psychoneuroendocrinology.

[CR42] Lyte M (2011). Probiotics function mechanistically as delivery vehicles for neuroactive compounds: microbial endocrinology in the design and use of probiotics. Bioessays.

[CR43] Mannie ZN, Harmer CJ, Cowen PJ (2007). Increased waking salivary cortisol levels in young people at familial risk of depression. Am J Psychiatry.

[CR44] Mayer EA (2011). Gut feelings: the emerging biology of gut–brain communication. Nat Rev Neurosci.

[CR45] Messaoudi M, Lalonde R, Violle N (2011). Assessment of psychotropic-like properties of a probiotic formulation (Lactobacillus helveticus R0052 and Bifidobacterium longum R0175) in rats and human subjects. Br J Nutr.

[CR46] Mogg K, Bradley BP (1998). A cognitive-motivational analysis of anxiety. Behav Res Ther.

[CR47] Mogg K, Bradley BP, Hallowell N (1994). Attentional bias to threat: roles of trait anxiety, stressful events, and awareness. Q J Exp Psychol Sect A.

[CR48] Murphy SE, Norbury R, O’Sullivan U (2009). Effect of a single dose of citalopram on amygdala response to emotional faces. Br J Psychiatry.

[CR49] Murphy SE, Yiend J, Lester KJ (2009). Short-term serotonergic but not noradrenergic antidepressant administration reduces attentional vigilance to threat in healthy volunteers. Int J Neuropsychopharmacol Off Sci J Coll Int Neuropsychopharmacologicum (CINP).

[CR50] Nelson H (1982). The National Adult Reading Test (NART): test manual.

[CR51] Pariante CM, Lightman SL (2008). The HPA axis in major depression: classical theories and new developments. Trends Neurosci.

[CR52] Pizzagalli DA (2010). Frontocingulate dysfunction in depression: toward biomarkers of treatment response. Neuropsychopharmacology.

[CR53] Pruessner JC, Wolf OT, Hellhammer DH (1997). Free cortisol levels after awakening: a reliable biological marker for the assessment of adrenocortical activity. Life Sci.

[CR54] Rao AV, Bested AC, Beaulne TM (2009). A randomized, double-blind, placebo-controlled pilot study of a probiotic in emotional symptoms of chronic fatigue syndrome. Gut Pathog.

[CR55] Rhee SH, Pothoulakis C, Mayer EA (2009). Principles and clinical implications of the brain–gut–enteric microbiota axis. Nat Rev Gastroenterol Hepatol.

[CR56] Savignac HM, Corona G, Mills H (2013). Prebiotic feeding elevates central brain derived neurotrophic factor, N-methyl-d-aspartate receptor subunits and d-serine. Neurochem Int.

[CR57] Schlotz W, Yim IS, Zoccola PM (2011). The perceived stress reactivity scale: measurement invariance, stability, and validity in three countries. Psychol Assess.

[CR58] Sheline YI, Barch DM, Donnelly JM (2001). Increased amygdala response to masked emotional faces in depressed subjects resolves with antidepressant treatment: an fMRI study. Biol Psychiatry.

[CR59] Spielberger C, Gorsuch R, Lushene R (1970). Manual for the State-Trait Anxiety Inventory (STAI).

[CR60] Sudo N, Chida Y, Aiba Y (2004). Postnatal microbial colonization programs the hypothalamic–pituitary–adrenal system for stress response in mice. J Physiol.

[CR61] Tillisch K, Labus J, Kilpatrick L (2013). Consumption of fermented milk product with probiotic modulates brain activity. Gastroenterology.

[CR62] Vulevic J, Drakoularakou A, Yaqoob P, et al (2008) Modulation of the fecal microflora profile and immune function by a novel trans-galactooligosaccharide mixture (B-GOS) in healthy elderly volunteers. Am J Clin Nutr 88:1438–1446 10.3945/ajcn.2008.26242 18996881

[CR63] Watson D, Clark L, Tellegen A (1988). Development and validation of brief measures of positive and negative affect: the Positive and Negative Affect Schedule scales. J Pers Soc Psychol.

[CR64] Wolfram M, Bellingrath S, Kudielka BM (2011). The cortisol awakening response (CAR) across the female menstrual cycle. Psychoneuroendocrinology.

[CR65] Young AW, Rowland D, Calder AJ, et al (1997) Facial expression megamix: tests of dimensional and category accounts of emotion recognition. Cognition 63:271–313 10.1016/s0010-0277(97)00003-6 9265872

